# Pressure-Volume Work for Metastable Liquid and Solid at Zero Pressure

**DOI:** 10.3390/e20050338

**Published:** 2018-05-03

**Authors:** Attila R. Imre, Krzysztof W. Wojciechowski, Gábor Györke, Axel Groniewsky, Jakub. W. Narojczyk

**Affiliations:** 1 Thermohydraulics Department, MTA Centre for Energy Research, P.O. Box 49, 1525 Budapest, Hungary; 2 Budapest University of Technology and Economics, Department of Energy Engineering, Muegyetem rkp. 3, H-1111 Budapest, Hungary; 3 Institute of Molecular Physics, Polish Academy of Sciences, ul. M. Smoluchowskiego 17, PL-60-179 Poznan, Poland; 4 President Stanisław Wojciechowski State University of Applied Sciences, Nowy Swiat 4, 62-800 Kalisz, Poland

**Keywords:** adiabatic, aergiatic, isobaric, heat exchange, metastate, metastability, negative pressure, spinodal

## Abstract

Unlike with gases, for liquids and solids the pressure of a system can be not only positive, but also negative, or even zero. Upon isobaric heat exchange (heating or cooling) at *p* = 0, the volume work (*p-V*) should be zero, assuming the general validity of traditional *δW = dW_p_ = −pdV* equality. This means that at zero pressure, a special process can be realized; a macroscopic change of volume achieved by isobaric heating/cooling without any work done by the system on its surroundings or by the surroundings on the system. A neologism is proposed for these *dW_p_* = 0 (and in general, also for non-trivial *δW* = 0 and *W* = 0) processes: “aergiatic” (from Greek: *Ἀεργία*, “inactivity”). In this way, two phenomenologically similar processes—adiabatic without any heat exchange, and aergiatic without any work—would have matching, but well-distinguishable terms.

## 1. Introduction

**Figure 1:**
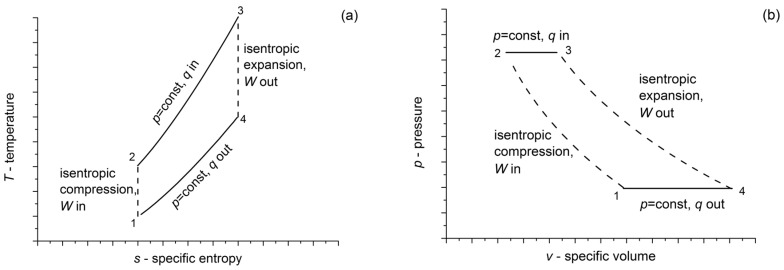
Schematic representation of a Joule-Brayton thermodynamic cycle on temperature-entropy (**a**) and pressure-volume space (**b**), consisting an isentropic (reversible adiabatic) compression, an isobaric heating an isentropic expansion and an isobaric cooling.

Isobaric heating/cooling is one of the basic theoretical processes of thermodynamics, as well as of energy engineering. Some of the basic thermodynamic cycles—like Rankine or Joule-Brayton—comprise one or more steps, when heating/cooling happens at constant pressure. A schematic diagram of the Joule-Brayton cycle can be seen in Figure 1. This cycle is frequently used in gas turbines to convert heat to movement, and then to electricity. This has an isentropic (reverse and adiabatic) compression (1-2), then an isobaric heat intake (2-3), an isentropic expansion (3-4) and an isobaric heat rejection (cooling) (4-1), before starting the cycle again. During the isobaric heat exchange, temperature, as well as volume, change simultaneously, i.e., part of the heat can be converted into kinetic energy. Although in most cases Joule-Brayton cycles are open to a given environment (using an open internal combustion chamber), it is also possible for heating and cooling to apply heat exchangers, making a thermodynamically closed system.

In a similar manner, isobaric heating for condensed matter (liquids as well as solids) is also a common process. In the case of isobaric heating (common heating of any object on atmospheric pressure, for example), the volume change can be described by the isobaric heat expansion. Although the volume effect on heating is smaller for gases that that of liquids and solids, the description of the process is the same. 

In this paper, we would like to address the problem that occurs in condensed matters where, under some circumstances, the absolute scalar pressure of the system can be non-positive, i.e., *p* ≤ 0 [1,2,3]); a special process can exist in an isobaric heat exchange (heating or cooling) at *p* = 0. During this process, a finite volume change can occur, while the pressure-volume work remains at zero. Although *p* = 0 states are considered as “common” states for condensed matters (unlike for gases, where *p* = 0 is the absolute lower limit for the existence of any gaseous state), the existence of these processes shows that in some sense, these states are special.

## 2. Isobaric Heat Exchange and the First Law of Thermodynamics 

The total change of internal energy for a closed system can be written as:

*dU* = *δQ* + *δW* (1)

by the First Law of Thermodynamics (in the form recommended by the IUPAC [4,5]), where *δQ* and *δW* are the heat and work of the infinitesimal process. During a thermodynamic cycle—where the initial and end states are the same—the total change of the internal energy will be zero, but some of the heat can be converted into work or vice versa. 

At various steps of the cycle, for example during the step of isobaric heating, the work—which is called volume, or *p-V* work in this case—can be calculated as:

(2) $$W=-\overset{V_{2}}{\underset{V_{1}}{\int }}p\left(V\right)dV=-p*\Delta V$$

where *V*_1_ and *V*_2_ are the volumes of the initial and final state respectively. The second equality holds only for isobaric processes, where *p* is the constant (volume-independent) pressure of the process, and Δ*V* is the volume difference between the initial and final states. The negative sign is due to the fact that during compression (negative Δ*V*), we transfer energy to the system; therefore, the work done on the system should be positive. In general, *p-V* work is the work done by the system on the environment during expansion, or that done by the environment on the system upon compression. One should be aware that the system and the environment are in equilibrium, i.e., the internal and external pressures are equal. In this way, the volume change is not the result of a pressure change. When there is an isobaric heat exchange, it is caused by thermal expansion or contraction by isobaric heating or cooling.

Although work—just like heat—is not a state function, during isobaric processes (as well as during any other simple process with a fixed path in the field of state variables), the infinitesimal change of work can be written as *dW_p_*, instead of the path-dependent *δW*:

(3) $${\delta W|}_{p}\equiv dW_{p}$$

Hereafter this nomenclature will be used, although in Appendix A, generalized (not necessarily isobaric) *δW* also will be used.

In gases, where only *p* > 0 states exist and the system always expands upon heating and contracts upon cooling, the previous statement (that compression corresponds to a positive work, done on the system) seems to be appropriate. For condensed matters (liquids, solids and glasses) however, where *p* ≤ 0 states [1,2,3], or materials with negative thermal expansion (like water below 4 °C) exist, the determination of the proper +/− sign requires some care. Doing a heat exchange at negative pressure, *pΔV* might lead to contradictions in the logic applied in the positive pressure range. The principle is also seems to be violated when negative thermal expansion is considered, since the volume change (Δ*V*) will be negative upon heating, and positive upon cooling, differing from the behavior of “normal” materials. These problems have already been discussed by a few authors, with varying levels of success [6,7,8,9,10,11]; in this paper, we are going to mention them only when there is some connection with our problem, which is the proper calculation of work upon isobaric heating/cooling of liquids at *p* = 0.

According to Equation (2), upon isobaric heating or cooling in zero pressure, *p-V* work remains zero at any stage, defining a unique process. While adiabatic processes (*Q* = 0) are well known, these “work-free” processes were ignored—although they can cause various theoretical problems. Obviously, there are other cases where the path-dependent and path-independent types of infinitesimal work (*δW* or *dW_p_*) or their finite counterpart (*W*) will be zero; some of them are mentioned in the Appendix A.

**Figure 2:**
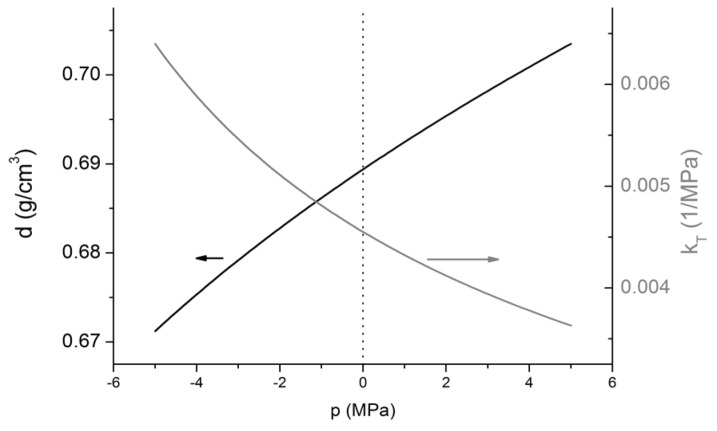
Mass-density (black, left y-axis) and isothermal compressibility (grey, right y-axis) of liquid (metastable) water at 573.15 K (300 °C), calculated by IAPWS equation of states [21], demonstrating the lack of any anomaly at *p* = 0 value. At this temperature, stable liquid water can exist only above 8.588 MPa.

When liquids or solids are in equilibrium with vapor, *p* = 0 pressure is a special value, because neither sublimation pressure, nor vapor pressure can reach this value; rather, it can only be approached [1,2,3,12,13,14]. When only condensed matter is present in a closed system (the internal part of an open system can also be considered to be a closed one) without any equilibrium vapor, then the transition from negative to positive pressures, and crossing *p* = 0 within a process, or even a complete process where pressure is zero, can be realized theoretically, as well as experimentally. For example, while crossing the *p* = 0 value in a liquid upon isochoric cooling (see, for example, isochoric cooling experiments in liquid inclusions [15,16,17,18,19]), one cannot see any anomalous properties in the material. Material properties change smoothly, without extrema, inflection, asymptote or discontinuity. Figure 2 demonstrates the density (left y-axis, black line) and isothermal compressibility (right y-axis, grey line) of liquid water at 573.15 K, calculated by ThermoC [20], using IAPWS equation of states [21]. Hence, at this temperature, stable liquid water can exist only above 8.588 MPa; only metastable liquid states are represented here. It can be seen that the *p* = 0 value is crossed by both lines without showing any characteristic change in either property. In general, this is true for any frequently observed material properties [2,22]. Due to this smooth transition, one can conclude that *p* = 0 states are not special among the other metastable states.

In contrast to the non-specialty of *p* = 0 for condensed matters, a common opinion is that for liquids, there are two special pressure values for a given temperature: (i) the positive binodal pressure (equilibrium between liquid and vapor phases; boiling/condensation should occur here), and (ii) the spinodal pressure (the stability limit, where stretched liquid must boil by explosive spinodal transition; depending on the temperature, it can be positive, negative or even zero) [22]. Therefore, the existence of a special work-free process at *p* = 0, where nothing special was expected, creates a surprise for understanding the physics of metastable liquids.

Concerning solids, *p* = 0 states are simpler in some sense, but more difficult in others. At *p* = 0, solids do not seem to be metastable; they can stay in this condition infinitely (see, for example, a solid particle in space at almost zero pressure). But for solids, application of scalar pressure instead of a tensorial stress or pressure tensor needs special conditions [22]. An additional problem is related to the equation of states. For several liquids, equations of states extendable even to moderately metastable states are available (see for example for water IAPWS equation of states [21]). They can be used down to moderately negative pressures [23,24,25,26]. For solids, however, equations of states for *p* ≤ 0 pressures are very rare [14,27,28,29]. This is the reason why, in the following example, a “virtual experiment” uses water as example.

## 3. Isobaric Heating/Cooling of Water at Zero Pressure

### 3.1. Virtual Experiment

Although the existence of a *p* = 0 isobaric process might sound strange at first, one should accept the fact that in metastable liquids, *p* = 0 states are as real as any other *p* = constant states (like *p* = 0.1 MPa). In the same way, we have to accept, that the existence of a process through these *p* = 0 states are as plausible as the existence of any other isobaric process on any other constant pressure value (for example, the heating of overheated, metastable liquid water from 101 to 106 °C under constant, atmospheric pressure).

**Figure 3:**
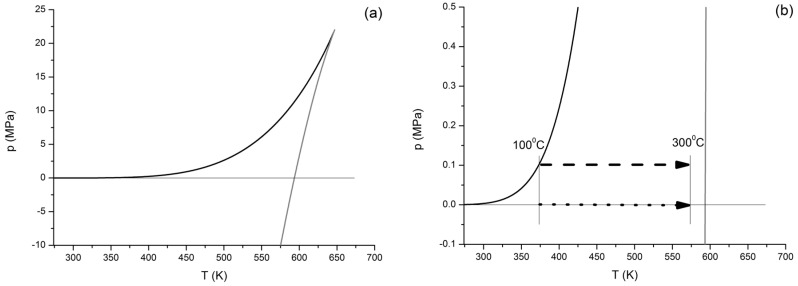
(**a**) Equilibrium vapor pressure line (black) and liquid-vapor stability line (grey) for water by IAPWS equation of states. (**b**) Representation of isobaric heating of metastable liquid water from 100 to 300 °C at atmospheric pressure (dashed) and at *p* = 0 (dotted).

Between its triple point temperature and critical temperature, water can be stable liquid at pressures not smaller than its equilibrium vapor pressure (also called saturation pressure). The vapor pressure is always positive; by dropping the pressure below this value at a given temperature, the water should boil. While boiling is the most probable phenomenon which is expected in this case, occasionally one can see that the water remains in a metastable liquid state. In Figure 3a, one can see the equilibrium vapor pressure line (black), as well as the so-called spinodal line (grey), extending from the critical point to the low, and eventually to the negative, pressure region. This spinodal is also called the liquid-vapor spinodal, indicating that the corresponding metastable phase is liquid, and that instability turns it partly or fully to vapor. The vapor pressure line separates the stable liquid and vapor states, but it does not say anything about metastable ones. Between these two lines, water might exist in a metastable liquid state [1,2,3]. It should be mentioned here that a similar spinodal line for undercooled vapor also exists (that would be the vapor-liquid spinodal), located on the other side of the vapor pressure curve; however, for the sake of clarity, it is not shown here. All metastable states can be reached by depressurization or isotropic stretching of high-pressure stable liquids; metastable states with *p* > 0 can be also reached by the overheating of low temperature stable liquids. More details about this special condition—including the “know-how” to reach them—can be seen is several sources [1,2,3,12,30,31].

The part of the spinodal curve where *p* ≥ 0 is called the limit of superheat or overheat. Even in high school physics or chemistry laboratories, one can see very pure water heated up to 102–105 °C under atmospheric pressure. Under special laboratory condition, this overheating (still at 1 bar pressure) can go over 300 °C. In Figure 3b (which is the magnified part of Figure 3a around *p* = 0) one can see two processes, marked by dashed and dotted lines. The dashed line represents an isobaric heating process of metastable water at atmospheric pressure (taken as 0.1 MPa) between 100 and 300 °C, while the dotted one represents a similar process at *p* = 0 pressure. All calculations for this virtual experiment were done by ThermoC [20]; this is probably the only widely available free program for thermodynamic property calculations which can handle metastable states.

Some more details about the way to have *p* = 0 isobaric heat exchange in a system of two immiscible liquids, while keeping the pressure at zero value, can be seen in Appendix B.

### 3.2. Accepting Isobaric p = 0 Processes as Special Ones

Concerning the 1 bar process, the initial density (at 1 bar and 100 °C) is 958.35 kg/m^3^, changing to 689.71 kg/m^3^ (still liquid, although highly metastable) when 300 °C is reached. Assuming 1 m^3^ initial volume, the final volume will be 1.3895 m^3^, giving Δ*V* = 0.3895 m^3^. Using Equation (2), the work is *W* = −0.1 × 10^6^ × 0.3896 = −38.96 kJ. 

Concerning the 0 bar process, although the volume change will be almost the same (the initial density for metastable liquid water at 0 bar and 100 °C is 958.30 kg/m^3^, changing to 689.39 kg/m^3^ reaching 300 °C; also assuming 1 m^3^ initial volume, the final volume will be 1.3901 m^3^, giving Δ*V* = 0.3901 m^3^), the pressure and work will be zero (*W* = 0). From this huge difference (−38.96 kJ vs. 0 kJ), it can be seen that we are not talking about minute values, but macroscopic, well-measurable quantities; even for 1 L of water, the “vanishing” work is 39.96 J. Starting the same process from the solid-liquid transition (around 0 °C), the volume change can be even bigger.

Therefore, one has to accept that although a *p* = 0 state might not be unique, there is at least one process (isobaric *p* = 0 heat exchange) which has this special characteristic at this pressure, making the state itself somehow unique.

### 3.3. Introduction of a New Pressure Scale

We can assume that while Equation (2) is valid for gases, it is not appropriate for condensed matters. Although this idea seems to be quite far-fetched, one should address this issue as well.

We should remember that in the work of Gay-Lussac, studying *p/T* in isochoric and *V/T* in isobaric systems, the linearity was obvious, but not the proportionality. The reason was simple: the temperature scale (Celsius) used by Gay-Lussac had an incorrect zero point. Shifting the zero point to the correct place (Kelvin-scale) would make the explanation of the Gay-Lussac experiments much simpler. 

Although the zero point of the pressure scale comes in a straightforward way from mechanics or from gas dynamics, there are some examples related to metastates where a properly shifted pressure scale would give a more accurate, or at a least very simple description, for some phenomena. Rzoska and his co-workers [32,33] introduced a new pressure-scale (marked here as *π* instead of *p*), describing the pressure dependence of several properties, including glass transition temperature, dielectric permittivity, etc. The peculiarity of this new scale was the monotonously temperature-dependent zero point. Although initially the new zero point (for a given temperature) was only a fitting parameter, later it turned out that it might be close, or even equal to the liquid-vapor spinodal pressure. This means that for a given liquid state, the pressure governing various properties of liquids is not the distance on the pressure scale from zero pressure, but the distance at a given temperature; the ultimate existence limit of the liquid state is:

(4) $$\pi \left(T\right)=p-p_{L-V}\left(T\right),$$

where *p* is the actual pressure and *p_L-V_(T)* is the liquid-vapor spinodal at the given temperature. Since this new quantity marks a distance, it might therefore be called “pressure distance” to distinguish it from the traditional pressure.

**Figure 4:**
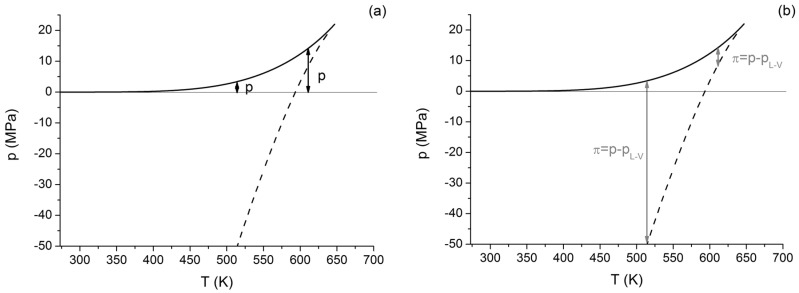
Comparison of real pressures marked as *p* (black arrows, (**a**)) with the pressure distance from the liquid-vapor spinodal, marked as *π* (grey arrows, (**b**)).

The difference between the two definitions for liquids can be seen in Figure 4. In Figure 4a,b, one can see the regular pressure (*p*) and the new “pressure distance” (*π*) at two different temperatures. One can see that, depending on the temperature, *π* can be either smaller or bigger than *p*; even equality can be obtained in the temperature, where the spinodal crosses *p* = 0.

One should be aware, that this new pressure scale would also solve some problems related to work under negative pressure, as raised by Stepanov [9,10,11], if the new pressure value (*π*) is always non-negative (or even positive, accepting the physical unattainability of the spinodal).

Concerning solids, they also have some kind of stability limit (for example limiting deepest negative pressure for their existence), but the exact nature of this limit is still not clear [13,27,28,29]. For liquids, approaching the stability limit (spinodal), one can see bigger and bigger density fluctuations, reaching even density values characteristic of gases. For solids, it seems to be unrealistic to expect the same behavior. One can say that the lower pressure limit for the existence of solids is probably a solid/vapor-type stability limit, rather than a solid/liquid one, since the estimated stability limits for various solids are far below the pressures where liquid in any form can exist. But even without knowing the nature of this stability limit, mathematically one can also define a *π* for solids, where:

(5) $$\pi \left(T\right)=p-p_{s}\left(T\right)$$

where *p_s_* would be the generalized, ultimate stability limit for solids at a given temperature. Because all pressure-related quantities are scalar here, this method would probably fail for anisotropic solids, where one might expect direction-dependent stability limits. Also, it would fail where pressure tensor should be used, instead of scalar pressure.

For gases, it can be clearly seen that the two scales—the traditional pressure scale and the new “pressure distance” scale—are identical (*p* ≡ *π*), since for gases, the lowermost possible pressure is zero; negative pressures do not exist for them and the existing thermodynamic stability limit - vapor-liquid spinodal—is an upper bound, instead of a lower one [1,2].

The work based on the “pressure distance” could be calculated as:

(6) $$W_{\pi }=-\overset{V_{2}}{\underset{V_{1}}{\int }}\pi \left(V\right)dV=-\pi *\Delta V,$$

where the subscript *π* would mark the difference from the traditionally calculated work in Equation (2). 

Although accepting Equation (6) as the correct calculation for volume-works would solve some problems, it would raise at least two other ones. First of all, introduction of a new pressure scale would turn classical thermodynamics upside down. For example, any traditional *p* = constant heating/cooling would not be “isobaric” for the new *π* “pressure distance” scale, because *p_L-V_* as well as *p_s_* are temperature-dependent quantities.

Secondly, there is a technical problem. *p-V* work for liquids are usually neglected in thermodynamic cycles, because it is much smaller than its counterpart in vapor. Technically, it is manifested as a very minor loss, together with other losses (caused by friction, heat loss, etc.). Since the difference between *p* and *π* can be huge at low, very low and very high temperatures, the difference between works calculated by the two methods (Equations (2) and (6)) would be significant; certainly, it would be detectable during experiments or during everyday industrial processes. In Table 1, one can compare the *p-V* works for given mass of water done on liquid (subscript *l*) and vapor (subscript *v*) at two different pressures (0.1 and 5 MPa) in a Δ*T* = 0.2 K heating process. The initial temperature for liquid and vapor is 0.8 K below and 0.6 K above the boiling point for the given pressure, respectively. In this way, the average distance on the temperature scale will be the same (0.7 K). *p-V* work for liquid will be given by the traditional Equation (2) and by the “pressure distance” Equation (6) method for the liquid state, while for vapor, only the traditional method Equation (2) is used (the results using the two methods are identical for gases). The methods are marked by the letter *p* or *π* in bracket. In the last two columns, the ratio of the *p-V* works done by the liquids and gases can be seen, calculated using both methods.

Using the traditional calculation method—used presently by process- or energy-engineers—in low pressure, the *p-V* work of liquid is negligible compared to the *p-V* work of gas. Even at higher pressures, the ratio is slightly above 1%, giving only a very small—probably negligible—contribution for energy balance. In industrial/technical processes, efficiencies (given in %, usually a few tens of % for a typical power plant, like 30–40%) are usually given in one-digit accuracy; therefore, processes contributing only a few tenths of %—like 0.1–0.5%—of the total energy balance can be easily neglected. On the other hand, the “pressure distance” method gives ratio which is almost an order of magnitude bigger, between the *p-V* works of steam and liquid; this would not be negligible in the energy balance. The situation at atmospheric pressure is much worse; while by Equation (2), the *p-V* work of liquid is absolutely negligible compared to the similar work of steam, by the “pressure distance” method (Equation (6)) the ratio is around 25%, giving a non-negligible liquid contribution. Since steam engines have been used for more than two centuries while the traditional method has been used to calculate *p-V* work all the time, one can conclude that a big loss caused by the *p-V* work of liquid should not remain hidden. Therefore, this big loss does not exist. In this way, one can see, that the proper calculation of the *p-V* work can be done using Equation (2), rather than Equation (6). Although the introduction of the “pressure distance” could be used to explain various pressure-related dependencies, in this case, it is definitely not a good solution.

## 4. Discussion

### 4.1. Aergiatic Processes

Based on the experimental evidence shown in the previous section, one can conclude, that *p-V* work in condensed matters at *p* = 0 pressure has to be calculated by Equation (2), giving *dW* = 0 as well as *W* = 0. This means that at *p* = 0 pressure, volume can be changed by a finite Δ*V* value by isobaric heating/cooling through a finite Δ*T* range without any work done by the system on its surroundings, or by the surroundings on the system. 

These processes are phenomenologically similar to adiabatic processes (*δQ* = 0), and although they are probably less important, they still deserve a name of their own. Since in these processes, the works is always zero, our suggestion for a name is “aergiatic”, originating from “Aergia” (Greek: *Ἀεργία*, “inactivity”); a minor goddess (Daimona) in Greek mythology, a personification of idleness, indolence and laziness. We believe, that this name matches its heat-related counterpart (adiabatic) quite well.

Some generalization for aergiatic processes can be seen in the Appendix A.

### 4.2. p = 0 States vs. p = 0 Processes

At the end of this paper, still one controversy remained unsolved, namely that while the *p* = 0 itself is not special value, there is at least one process along *p* = 0 which seems to be special (*dW_p_* = 0). It is a little bit similar to the liquid-vapor and vapor-liquid stability lines [1,2,3,12]. By heating or stretching a liquid, and hence by moving first into the stable and then metastable liquid regions, the liquid will not detect the vapor-liquid spinodal, but only its counterpart, the liquid-vapor one. The opposite is also true: coming from the stable vapor side, only the vapor-liquid stability line will have any meaning upon the particular processes; the liquid-vapor one remains undetected. In this way, some of the special lines or values will be direction-dependent. The situation is the same with *p* = 0 values. Generally, upon crossing or touching *p* = 0, one cannot detect any peculiarity, except in two “directions” in a pressure-temperature diagram: by isobaric (*p* = 0) heating or by isobaric (*p* = 0) cooling.

It might be useful to mention that although *p* = 0 states can be observed experimentally, there is some equation of state—sufficient for stable states—which are not able to handle *p* ≤ 0 values, like the Dieterici equation of state [34].

## 5. Summary

Unlike for gases, for condensed matters (liquids, solids and glasses) the pressure of the system can be not only positive, but also negative or even zero. At *p* ≤ 0 conditions, for liquids and glasses (and maybe even for solids), the normal phase will be metastable (might boil or vaporize spontaneously at any time), therefore these states are referred as metastates.

Upon isobaric heat exchange (heating or cooling) at *p* = 0 condition, the volume (*p-V*) work should be zero, assuming the general validity of the traditional *δW* ≡ *dW_p_* = *−pdV* equality. This means that there is a special condition (*p* = 0), where a special process can be realized: macroscopic changes of volume by isobaric heating/cooling without any work done by the system on its surroundings or by the surroundings on the system, i.e., *W* (as well as *dW_p_* and *δW*) is zero. An alternative way to calculate *p-V* work based on the distance on the pressure scale from the lowermost limit of existence of the given phase proved to be invalid.

Although by crossing *p* = 0 value, neither microscopic (molecular) nor macroscopic (for example phase transition) change can be seen in condensed matters, the *p* = 0 condition itself still seems to be a special, due to the existence of this *dW*_p_ = 0 process.

An name is proposed for these *dV* ≠ 0 but *dW_p_* = 0 (and in general, also for *W* = 0) processes. The name “aergiatic” processes—named after Aergia (Greek: *Ἀεργία*, “inactivity”), a minor goddess (daimona) in Greek mythology, a personification laziness, idleness, and indolence—seems to be a proper one. In this way, two phenomenologically similar processes—adiabatic without any heat exchange, and aergiatic without any work—would have matching, but well-distinguishable names.

## Figures and Tables

**Table 1:** Specific *p-V* works for liquids water and steam calculated by Equations (2) and (6) and their ratio.

*p* (MPa)	*W_l_(p)* (kJ/kg)	*W_l_(π)* (kJ/kg)	*W_g_(p)* (kJ/kg)	*W_l_(p)*/*Wg*	*W_l_(π)*/*Wg*
0.1	−0.0000156	−0.02544	−0.0983	0.000158	0.2589
5	−0.0028207	−0.02216	−0.1909	0.0148	0.1161

**Figure A1:**
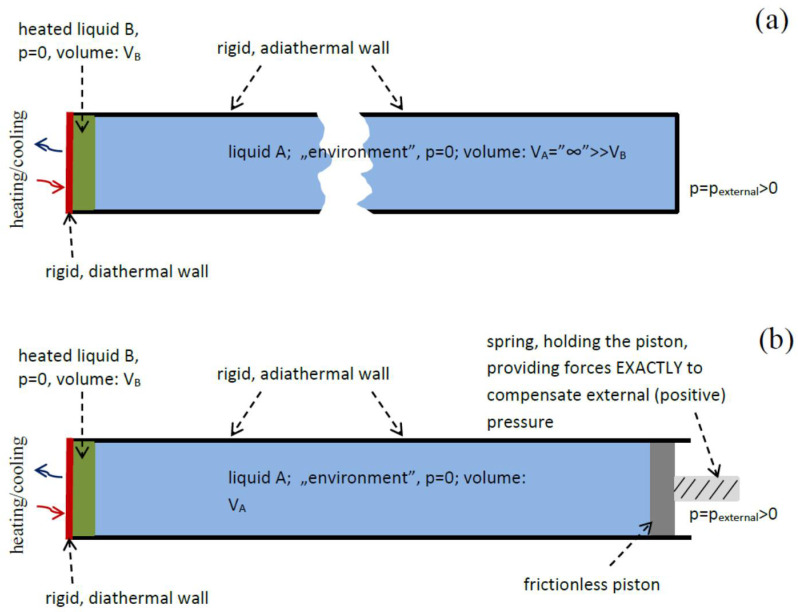
Potential experimental realizations of *p* = 0 isobaric heat exchange in systems of two (A and B) liquids. (**a**) Idealized case with infinite volume second liquid phase (environment) (**b**) Realistic case with finite volume liquid B with tunable pressure.

## Appendix A

Obviously there are other cases, when the obtained work is zero during an isobaric heating/cooling within a non-zero temperature range. Since the work can be calculated as:

(A1) $$W\;=\;-\overset{V_{2}}{\underset{V_{1}}{\int }}pdV$$

it will be zero, when *V* remain constant (*dV* = 0); this is a trivial solution. In this case, the density of the material will be constant and the isobaric heat expansion coefficient is zero in the observed temperature range. Having isobaric (*p* = constant) process, Equation (A1) can be replaced by:

(A2) $$W\;=\;-p\Delta V\;=\;-p\left(V_{2}-V_{1}\right).$$

Here, the equality of initial and final volumes (*V*_1_ and *V*_2_) also leads to zero work; this is not identical with the first case, because *dV* can be nonzero, but the positive and negative volume changes should be equal during the process. Also, *δW* (or *dW_p_*) is also non-zero, but after integration, *W* = 0. This second case (Equation (A2)) can be true for non-isobaric processes too, but for demonstration, the isobaric case is more straightforward. In similar manner, the free expansion (Joule expansion) of ideal gases can be also considered as a “trivial aergiatic” process; since the volume change of the total system upon the gas expansion is zero, therefore the work is also zero.

Experimentally *dV* = 0 case can be realized with composite materials (mixture of two or more materials where average heat expansion coefficient is zero) or with materials with special microscopic structure, while the second case can be realized by materials with special density-temperature dependence, showing maximum (like for water around 4 °C) or minimum.

Both of the cases described in the Appendixs can be realized in non-isobaric processes too. Under generalized (non-isobaric) conditions, the aergiatic process can be characterized by *δW* = 0 in the first—constant volume—case (*δ* is marking the infinitesimal change of a path-dependent function, like work). In the second case, the condition (*W* = 0) remains the same.

This is also a way to soften the definition of aergiatic processes. In the first case, when *dV* = 0, might be referred as “trivial aergiatic” process; in this case *dW_p_* = 0, *δW* = 0 and *W* = 0 equalities are true (obviously, *dW_p_* can be considered only during isobaric processes). In the second case, where Δ*V* = 0 can be referred as “extended aergiatic” process; in this case *dW_p_* ≠ 0, *δW* ≠ 0 and *W* = 0. For reminder, the process is strictly aergiatic, when *dW_p_* = 0 and *W* = 0, while *dV* ≠ 0 and Δ*V* ≠ 0.

It should be mentioned here, that in similar manner, one cannot define adiabatic processes by assuming zero temperature values. While negative temperature can exist [35,36], the *T* = 0 value itself cannot be reached, only approached asymptotically.

## Appendix B

Details about reaching pressures in liquids below the equilibrium vapor pressure (including absolute negative pressures or zero pressure) can be seen in Refs. [1,2,3,4,5,6,7,8,9,10,11,12,22,31] and will not be described here. In this part, we are focusing on the possible realization of isobaric heating/cooling.

While changing the temperature of a liquid (or even a solid) in equilibrium with its vapor phase, the pressure in the surrounding should be changed, due to the vaporization or sublimation. In this way, keeping any—even positive, like the atmospheric 0.1 MPa—pressure in constant value upon heating/cooling, either one needs some way to compensate the change or one should assume that the environment—in equilibrium with the system, i.e., having the same pressure—is practically infinite, therefore it can keep the initial constant pressure. This is the situation for example by heating any liquid in an open container; we assume that the few extra molecules evaporating from the liquid is negligible to the number of particles in the total atmosphere, therefore the pressure is assumed constant.

The situation is even more delicate, when the pressure of the isobar process is zero and has to be kept exactly on that value. In this way, even a minor change might invalidate “zero work” condition. Therefore we would like to describe two potential ways to realize experiments; they are shown in Figure A1a,b. Both “designs” consists a long cylinder, made from a rigid, adiathermal (no heat exchange allowed through it) material with zero thermal expansion coefficient. At one end, it is closed by a flat, rigid, but diathermal wall for heating/cooling. There are two immiscible liquids in the system (A and B); they can be really different ones (like oil/water) or they can be the same liquid, separated only by an imaginary interface. The pressure is zero in both liquids. It is important that the liquid or liquids are in contact only with the wall and with the other liquid; no vapor phase is in contact with them!

In first case (Figure A1a), the tube is “infinite” long and closed at the other end by a flat adiathermic rigid wall. This is a highly idealized situation, similar to the ones assumed for any other ideal isobaric heat exchange process. Upon heating/cooling, liquid A expands or contracts and therefore there would be some positive or negative work against the environment, represented by liquid B. The zero pressure is kept by the infinity of the volume of liquid B. One can see that although it is a good design for a “schematic” experiment, having real volume B as a finite value, pressure will always shifts from the initial value. This shift could cause problem, when strict zero value is required, therefore it has to be eliminated.

The second case (Figure A1b) attempts to solve this problem. Here the other end is closed by a piston, which can slide in the cylinder. Since the internal pressure is zero, while the external one is a non-zero positive one, the piston has to be hold in position by a spring. The force provided by the spring has to be equal to the force of external pressure on the piston all the time, even upon heating or cooling. In this way, the internal pressure can be kept exactly at zero. The spring/piston system can be replaced with other elements too. For example Hiro and his co-workers use a special cell to generate negative pressure [37,38], where part of one wall is replaced by a metal membrane; in that case the elastic forces of the membrane and the external pressure can compensate each other; the latter can be easily changed upon heating/cooling liquid A. Although they use that part to measure the internal/external pressure difference, it could be also used to regulate the internal pressure, therefore this method might be applicable to study really aergiatic processes.
